# Machine learning models based on magnetic resonance imaging for predicting Lymphovascular Invasion in Invasive Breast Cancer

**DOI:** 10.1371/journal.pone.0350085

**Published:** 2026-05-29

**Authors:** Hong Li, Jieling Huang, Jianning Hou, Xinxin Chen, Cheng Zhi, Zhiming Li

**Affiliations:** 1 Department of Radiology, the Second Affiliated Hospital, Guangzhou Medical University, Guangzhou, Guangdong, China; 2 Department of Radiology, Guangzhou Geriatric Hospital, Guangzhou, Guangdong, China; 3 Department of Radiology, Guangzhou Women And Children’s Medical Center, Guangzhou, China; 4 Department of Breast surgery, the Second Affiliated Hospital, Guangzhou Medical University,‌‌ Guangzhou, Guangdong, China; 5 Department of Pathology, the Second Affiliated Hospital, Guangzhou Medical University, ‌‌Guangzhou, Guangdong, China; Hokkaido University: Hokkaido Daigaku, JAPAN

## Abstract

**Objectives:**

Treatment strategies for invasive breast cancer require accurate lymphovascular invasion (LVI) predictions. This study aimed to investigate the feasibility and effectiveness of delta radiomics signature based on dynamic contrast-enhanced magnetic resonance imaging (DCE-MRI) and radiomics signature based on T2-weighted fat suppressed imaging(T2FS) for assessing LVI in invasive breast cancer.

**Materials and methods:**

A total of 166 patients with resectable invasive breast cancer who underwent preoperative DCE-MRI and T2FS from July 10, 2020 to December 31, 2023 were enrolled. Radiomics features were extracted from pre-contrast phase (RF_pre_), second post-contrast phase (RF_post_), and Delta radiomics features (RF_Delta_) calculated as RF_post_ minus RF_pre_. Then four radiomics signatures (RS_T2_, RS_pre_, RS_post_, RS_Delta_) were further developed based on the Random Forest model for RF_T2_, RF_pre_, RF_post_ and RF_Delta_, respectively. The predictive performance of all signatures was evaluated by receiver operating characteristic (ROC) analysis, with accuracy and area under the curve (AUC) as the main quantitative metrics.

**Results:**

In the test set, RS_Delta_ (10 features) achieved the highest accuracy of 0.717 and an AUC of 0.764; RS_post_ (8 features) had an accuracy of 0.565 and an AUC of 0.610; RS_pre_ (7 features) and RS_T2_ (6 features) both showed an accuracy of 0.565 with AUCs of 0.535 and 0.662, respectively. Statistical differences were observed in predictive performance between RS_Delta_ and RS_pre_, RS_post_ (both *p* < 0.05), while no significant difference was found between RS_Delta_ and RS_T2_ (*p* = 0.239).

**Conclusion:**

RS_T2_, RS_pre_, RS_post_ and RS_Delta_ are all feasible for predicting LVI in invasive breast cancer, and RS_Delta_ outperforms the other three radiomics signatures, which can serve as a potential non-invasive imaging biomarker for LVI prediction in clinical practice.

## Introduction

Breast cancer has emerged as one of the most prevalent malignancy cancers among women worldwide, surpassing lung cancer in incidence in 2020 [1]. This significant trend underscores the urgent need for enhanced awareness, prevention, and individualized treatment options for the disease [2–5]. Though the 10-year survival rate of breast cancer patients is high [6]，the recurrence rate remains 10–15% within 5 years of diagnosis [7–8]. In recent years, numerous studies have focused on predicting axillary lymph node(ALN) metastasis [9–10], disease-free survival [11], and recurrence-free survival [12] after surgery in breast cancer, all of which aim to optimize prognostic assessment and treatment decision-making.

Lymphovascular invasion (LVI), a pathological feature characterized by the presence of cancer cells within lymphatic or blood vessels, is a crucial prognostic factor closely associated with disease recurrence and poor survival outcomes. As an important histopathological prognostic feature besides histological grade [13], LVI is correlated with the proliferative activity marker Ki-67 [14] and nodal status [15]. Accurate preoperative prediction of LVI is essential for risk stratification, rational treatment planning, and precise prognostic evaluation of breast cancer patients. Traditional histopathological examination is the gold standard for LVI diagnosis but is limited by its invasiveness, as the result is only available after surgical resection. Therefore, the development of noninvasive imaging techniques for preoperative LVI prediction is highly needed in clinical practice.

Dynamic contrast-enhanced MRI (DCE-MRI), a functional imaging technique, captures temporal changes in tissue vasculature following contrast agent administration, thus providing unique insights into tumor biological characteristics. T2-weighted fat-suppressed imaging(T2FS) enhances the visualization of anatomical structures by suppressing fat signals, facilitating the identification of peritumoral changes associated with tumor aggressiveness. However, LVI status cannot be directly identified by visual assessment of DCE-MRI and T2FS images. Radiomics, which involves the extraction of high-dimensional quantitative features from medical images, has shown great promise in improving the diagnostic and prognostic accuracy of various malignancies. While several studies have explored radiomics for LVI prediction in breast cancer [16–19], research on delta-radiomics (based on the dynamic changes of radiomics features between pre- and post-contrast DCE-MRI) for this purpose remains rare. Thus, an in-depth exploration of the predictive efficacy of delta-radiomics for LVI is required to enhance clinical understanding and application.

In recent years, biomedical artificial intelligence has greatly advanced medical imaging diagnostics, especially in radiomics, model explainability, federated learning, and clinical translation [20–22]. Explainable AI (XAI) enhances the interpretability of machine learning models by quantifying feature contributions, which is essential for clinical trust. Federated learning enables collaborative model training without centralized data sharing, addressing privacy and multi‑center heterogeneity. Meanwhile, clinical translation requires rigorous validation, regulatory compliance, and real‑world workflow integration [23–25]. These advances provide a methodological foundation for the present study and support the clinical application of delta‑radiomics in LVI prediction.

This study aimed to systematically investigate the value of radiomics signatures derived from T2FS, single-phase DCE-MRI, and delta-radiomics of DCE-MRI for preoperative LVI prediction in invasive breast cancer. Specifically, we first constructed four radiomics signatures based on the Random Forest model for T2FS, pre-contrast, post-contrast, and delta-radiomics features, respectively. We further compared the predictive performance of these four signatures to screen the optimal one and explore the clinical application value of delta-radiomics in LVI prediction. Null hypothesis: Radiomics signatures based on T2FS, pre-contrast DCE-MRI, post-contrast DCE-MRI and delta-radiomics of DCE-MRI have no significant predictive value for LVI in invasive breast cancer, and there is no statistically significant difference in predictive efficacy among these four radiomics signatures.

## Materials and methods

### Ethics statement

This study was approved by the Institutional Ethics Review Board of the Second Affiliated Hospital, Guangzhou Medical University(IRB NO. 2024-hg-ks-50). Given its retrospective design, the requirement for informed consent was waived by the above-mentioned Institutional Ethics Review Board. The patients’ MR images used in this study were retrieved from the Picture Archiving and Communication System(PACS) after obtaining IRB approval on October 31, 2024. All procedures were performed in accordance with the Declaration of Helsinki.

### Study population

From July 10, 2020 to December 31, 2023, a total of 166 eligible patients with breast cancer were retrospectively recruited from the Second Affiliated Hospital, Guangzhou Medical University. Sample size was determined based on the established rule of thumb for radiomics and machine learning research (at least 5–10 cases per selected radiomics feature) combined with the clinical availability of invasive breast cancer patients with complete MRI data and pathological results in our center. This 166 eligible patients satisfies the recommended sample size for radiomic modeling for constructing radiomics signatures with 6–10 features in this study.

The inclusion criteria were: (i) pathologically confirmed invasive breast cancer; (ii) complete standard DCE-MRI and T2FS data acquired preoperatively; (iii) complete clinical and histopathological data available; (iv) mastectomy or lumpectomy performed within two weeks after MRI scans. The exclusion criteria were: (i) bilateral breast cancer; (ii) breast lesion biopsy performed before MRI scans; (iii) neoadjuvant chemotherapy administered before surgery; (iv) inadequate MRI image quality with artifacts or missing key imaging phases. The including patients were randomly divided into a training set and a test set at a ratio of 7:3 for model construction and external validation, respectively.

### MRI acquisition

MRI examinations were performed within two weeks before surgery for all patients. All the breast MRI scans were conducted on the patients in a prone posture using 1.5- or 3.0-MRI scanners (Siemens Amira 1.5T, Philips Achieva 1.5T, Philips Achieva 3.0T) equipped with an 7-channel or 8-channel surface breast coil.

The imaging sequences included T1-weighted images (T1WI), T2-weighted fat-suppressed images (T2FS), diffusion weighted imaging (DWI), and dynamic contrast-enhanced (DCE) images. A multi-phase contrast enhancement technology was used for T1-weighted DCE-MRI, including one pre-contrast and eight post-contrast phases, with each phase acquired for 60 seconds. After the pre-contrast scan, a gadodiamide contrast medium (0.2 mmol/kg) was intravenously injected at a rate of 3 ml/s using a high-power injector, followed by a 20 ml normal saline flush.

Detailed sequence parameters for T2FS and DCE-MRI on different scanners were as follows: (1) Philips Achieva 3.0T: T2FS (TR = 6033 ms, TE = 70 ms, FOV = 300 mm, slice thickness = 3 mm); DCE-MRI (TR = 4.5 ms, TE = 2.1 ms, FOV = 300 mm, slice thickness = 2.4 mm). (2) Siemens Amira 1.5T: T2FS (TR = 6210 ms, TE = 61 ms, FOV = 360 mm, slice thickness = 4.5 mm); DCE-MRI (TR = 4.91 ms, TE = 2.39 ms, FOV = 360 mm, slice thickness = 1.5 mm). (3) Philips Achieva 1.5T: T2FS (TR = 5000 ms, TE = 70 ms, FOV = 340 mm, slice thickness = 3 mm); DCE-MRI (TR = shortest, TE = shortest, FOV = 340 mm, slice thickness = 3 mm).

Two experienced radiologists with 20 and 12 years of professional expertise in breast imaging, respectively, independently assessed all MR images. Imaging features related to breast cancer were evaluated, including axillary lymph node (ALN) status, peritumoral edema (PE), internal enhancement patterns (IEP), and time-signal intensity curve (TIC): (1) MRI-reported ALN suspicious metastasis was defined as short axis >10 mm, long-to-short diameter ratio <1.6, eccentric cortical thickness >4 mm, and absence of fatty hilum [9]; (2) MRI-reported PE (MRI-PE) positivity was visually assessed by evaluating abnormal signal intensities around the tumor on T2FS [16]; (3) IEP was categorized as homogeneous, heterogeneous, and rim enhancement [16]; (4) TIC was classified into Type I (persistent enhancement), Type II (plateau enhancement), and Type III (washout enhancement). Discrepancies were resolved by consensus.

### Patient clinical and histopathological features

The clinical and histopathological features of all patients were collected as follows: age, menopausal status, tumor diameter, multifocality, axillary, Nottingham grade, Estrogen receptor(ER) status, Progesterone receptor(PR) status, human epidermal growth factor receptor 2 (HER2) status, Ki-67 proliferation index, and molecular subtypes. Histological analysis of surgical specimens was performed to confirm LVI status by a pathologist with more than 15 years of clinical experience, which involved examining hematoxylin and eosin-stained sections for carcinoma cells in endothelial-lined spaces in the peritumoral breast tissue [15].

ER, PR and HER2 expression were evaluated in accordance with the guidelines of the American Society of Clinical Oncology (ASCO) and United States and Canadian Academy of Pathology (USCAP) guidelines [26,27]. Hormone receptor (HR) was defined as positive, with≥1% of nuclear staining of ER or PR. The Ki-67 index was assessed with a cut-off value of 20% [28]. HER2 negativity was defined as immunohistochemical (IHC) staining grades 0 and 1 + , while HER2 positivity was confirmed by IHC staining grade 3 + ; fluorescence in situ hybridization (FISH) was performed for IHC grade 2 + cases to assess HER2 gene amplification [26,27]. Based on the receptor status, all the patients were subsequently classified into three subtypes as follows: (i) HR + /HER2 − ; (ii) HER2 + ; (iii) TN (triple-negative) [28].

### Workflow of study

The workflow of this study including (1) image segmentation, (2) radiomics feature extraction, (3) feature selection, (4) machine learning models construction, and (5) model performance evaluation.

### Tumor segmentation

T2FS and DCE-MRI sequences (pre-contrast and second post-contrast phases) of all breast cancer patients were collected in DICOM format. Images were first corrected for N4ITK distortion and resampled to uniform pixel size to reduce errors caused by different MRI scanners and parameters [29]. The 3D region of interest (ROI) of the tumor was manually delineated on the above images using the open-source ITK-SNAP software (Yushkevich PA, University of Pennsylvania, USA) [30]. The second post-contrast phase was selected for segmentation because the peak enhancement of the tumor lesion occurs within 60–120 seconds after contrast agent injection [9].

Tumor segmentation for all cases was initially performed by a radiologist with 12 years of expertise in breast imaging. Subsequently, 30 randomly selected cases were independently segmented by another radiologist with 20 years of breast imaging experience, and both radiologists were blinded to the patients’ clinical and histopathological information. To assess the reliability and reproducibility of segmentation, both the longest diameter and volume of the segmented tumor were measured by the two radiologists. The intra-class correlation coefficient (ICC) was used to quantify the inter-observer agreement of the measurements, and the ICC values of tumor long diameter and volume were 0.80–0.91 and 0.78–0.92, respectively. Only radiomics features with an ICC > 0.75, indicating satisfactory reproducibility, were selected for subsequent analysis.

### Radiomics features and their changes

For each 3D-ROI of breast cancer, 1012 radiomics features were extracted from T2FS (RF_T2_), pre-contrast DCE-MRI (RF_pre_), and second post-contrast DCE-MRI (RF_post_) using the PyRadiomics package (van Griethuysen et al., Erasmus MC Cancer Institute, Netherlands) [29]. The extracted features included seven categories: first-order statistics (n = 197), shape-based features (n = 14), gray level co-occurrence matrix (GLCM, n = 241), gray level run length matrix (GLRLM, n = 175), gray level size zone matrix (GLSZM, n = 176), neighboring gray tone difference matrix (NGTDM, n = 55), and gray level dependence matrix (GLDM, n = 154). Delta-radiomics features (RF_Delta_) were calculated to reflect the dynamic changes of radiomics features between pre- and post-contrast phases using the formula: RF_Delta_ = RF_post_ − RF_pre_.

### Feature selection

All extracted radiomics features were first subjected to z-score normalization to standardize their distribution and eliminate the influence of different measurement scales, which was a prerequisite for subsequent feature selection and machine learning model construction. Two complementary feature selection methods, minimum-Redundancy Maximum-Relevancy (mRMR) and least absolute shrinkage and selection operator (LASSO) regression, were combined to screen the optimal feature subset: (1) mRMR was first performed to eliminate redundant and irrelevant features by maximizing the relevance of features to LVI status and minimizing the mutual redundancy among features; (2) LASSO regression was then conducted to further select the most predictive features, with the regularization parameter λ tuned using 10-fold cross-validation, and the optimal λ corresponding to the minimal model bias was selected to determine the final feature subset and their corresponding coefficients.

To identify clinical and histopathological features associated with LVI status, univariate logistic regression analysis was performed, and features with a p-value < 0.05 were considered statistically significant and selected for subsequent multivariate logistic regression analysis.

### Machine learning models construction and validation

Four radiomics signatures (RS_T2_, RS_pre_, RS_post_, RS_Delta_) were developed based on RF_T2_, RF_pre_, RF_post_, and RF_Delta_ features, respectively. The predictive performance of the four radiomics signatures was evaluated in the training and test sets using receiver operating characteristic (ROC) analysis. The DeLong test was used to compare the discriminative ability of different radiomics signatures. The main evaluation metrics included accuracy (primary metric), AUC with 95% confidence intervals (CIs), sensitivity, specificity, positive predictive value (PPV), and negative predictive value (NPV) calculated for all metrics. To evaluate the clinical applicability and net benefit of the radiomics signatures, decision curve analysis (DCA) was performed to quantify the net benefit of the models across different threshold probabilities in both the training and test sets. Calibration curves were also used to assess the consistency between the predicted LVI probabilities and the actual observed LVI status of the models.

### Statistical analyses

All statistical analyses were performed using Python 3.10 (Python Software Foundation, Wilmington, Delaware, USA). Continuous variables were expressed as mean ± standard deviation or median with interquartile range according to distribution; categorical variables were reported as counts and percentages. The optimal cutoff for each radiomic feature was determined by the Youden index. Group comparisons used Student’s t‑test (normal distribution), Mann‑Whitney U test (non‑normal distribution), or chi‑square test (categorical variables). Inter‑observer agreement for segmentation was assessed by ICC, with ICC > 0.75 indicating good reproducibility. Model performance was evaluated using accuracy, AUC(95% CIs), sensitivity, specificity, PPV, and NPV. The DeLong test compared AUCs between models. A two‑sided p < 0.05 was considered statistically significant. Sample size was justified by the radiomics principle of 5–10 cases per feature, and the total 166 patients were sufficient for the 6–10 features in each signature. Training and test sets were randomly split at a 7:3 ratio with stratification to preserve class balance.

## Results

### Demographic and clinical profile of the study cohort

This study enrolled a total of 166 patients with invasive breast cancer, who were randomly divided into a training set (n = 120, 70%) and a test set (n = 46, 30%) at a 7:3 ratio. The training set was stratified into LVI-positive (n = 54, mean age: 51.52 ± 11.16 years) and LVI-negative (n = 66, mean age: 54.59 ± 11.17 years) groups, and the test set included 20 LVI-positive (mean age: 53.85 ± 14.71 years) and 26 LVI-negative (mean age: 52.31 ± 11.85 years) patients.

Descriptive statistics for all clinical and histopathological characteristics were presented as mean ± standard deviation (continuous variables) or n (percentage) (categorical variables), and group comparisons were performed using the Student’s t-test, Mann-Whitney U test or chi-square test. No statistically significant differences were observed in age, tumor diameter, menopausal status, multifocality, MRI-reported ALN status, MRI-PE, IEP, TIC type, Nottingham grade, ER, PR, HER2, Ki-67 status and molecular subtypes between the LVI-positive and LVI-negative groups in both the training and test sets (all *p* > 0.05, Table 1). The baseline data were well balanced, which ensured the reliability of subsequent model construction and validation.

**Table 1:** Comparison of Characteristics Between the Training and Test Sets.

Characteristics	Training set(n = 120)			Test set(n = 46)		
Negative (n = 66)	Positive (n = 54)	*p*	Negative (n = 26)	Positive (n = 20)	*p*
Age	54.59 ± 11.17	51.52 ± 11.16	0.136	52.31 ± 9.35	53.85 ± 14.71	0.667
Tumor diameter (mm)	32.94 ± 17.87	35.52 ± 15.48	0.085	28.54 ± 11.98	33.10 ± 12.62	0.206
Menopausal			0.782			1.0
No	32(56.06%)	28(51.85%)		12(46.15%)	9(45.00%)	
Yes	34(51.52%)	30(55.56%)		14(53.85%)	11(55.00%)	
Multifocality						0.413
No	37(56.06%)	28(51.85%)		16(61.54%)	9(45.00%)	
Yes	29(43.94%)	26(48.15%)		10(38.46%)	11(55.00%)	
MRI-ALN status			0.161			0.885
No	34(51.52%)	20(37.04%)		10(38.46%)	9(45.00%)	
Yes	32(48.48%)	34(62.96%)		16(61.54%)	11(55.00%)	
MRI-PE			0.377			0.526
No	43(65.15%)	30(55.56%)		12(46.15%)	12(60.00%)	
Yes	23(34.85%)	24(44.44%)		14(53.85%)	8(40.00%)	
IEP			0.717			0.336
Homogeneous	30(45.45%)	26(48.15%)		7(26.92%)	8(40.00%)	
Heterogeneous	34(51.52%)	25(46.30%)		17(65.38%)	12(60.00%)	
Rim enhancement	2(3.03%)	3(5.56%)		2(7.69%)	/	
TIC curves			0.533			0.892
Type I	8(12.12%)	8(14.81%)		2(7.69%)	1(5.00%)	
Type II	22(33.33%)	13(24.07%)		9(34.62%)	8(40.00%)	
Type III	36(54.55%)	33(61.11%)		15(57.69%)	11(55.00%)	
Nottingham grade			0.131			0.436
I	6(9.09%)	1(1.85%)		2(7.69%)	/	
II	30(45.45%)	32(59.26%)		10(38.46%)	9(45.00%)	
III	30(45.45%)	21(38.89%)		14(53.85%)	11(55.00%)	
ER			0.775			0.363
Negative	16(24.24%)	11(20.37%)		11(42.31%)	5(25.00%)	
positive	50(75.76%)	43(79.63%)		15(57.69%)	15(75.00%)	
PR			0.457			1.0
Negative	30(45.45%)	20(37.04%)		13(50.00%)	10(50.00%)	
Positive	36(54.55%)	34(62.96%)		13(50.00%)	10(50.00%)	
HER2			0.255			1.0
Negative	51(77.27%)	47(87.04%)		16(61.54%)	12(60.00%)	
Positive	15(22.73%)	7(12.96%)		10(38.46%)	8(40.00%)	
Ki-67			1.0			0.575
Negative	24(36.36%)	20(37.04%)		6(23.08%)	7(35.00%)	
Positive	42(63.64%)	34(62.96%)		20(76.92%)	13(65.00%)	
Cancer subtype						
HR + /HER2−	43(65.15%)	41(75.93%)	0.28	10(38.46%)	10(50.00%)	0.629
HER2+	15(22.73%)	7(12.96%)	0.255	10(38.46%)	8(40.00%)	1.0
TN	8(12.12%)	6(11.11%)	1.0	6(23.08%)	2(10.00%)	0.443

§MRI-PE, MRI-reported peritumoral edema; MRI-ALN, MRI axillary lymph nodes; IEP, Internal enhancement pattern; TIC, time-signal intensity; ER, oestrogen receptor; PR, progesterone receptor; HER2, human epidermal growth factor receptor 2.

### Selection of radiomics features

A total of 1012 radiomics features were extracted from T2FS, pre-contrast and post-contrast DCE-MRI phases, respectively, and delta-radiomics features (RF_Delta_) were further calculated as the difference between post- and pre-contrast features. All features were normalized by z-score, and features with an intra-class correlation coefficient (ICC) < 0.75 were excluded to ensure reproducibility.

Subsequently, mRMR was used to eliminate redundant and irrelevant features, and LASSO regression was further applied to screen the optimal feature subsets for each radiomics signature with the regularization parameter λ tuned by 10-fold cross-validation. The optimal λ values were determined as follows: RF_T2_ (λ = 0.0339, 6 features retained), RF_pre_ (λ = 0.0193, 7 features retained), RF_post_ (λ = 0.045, 8 features retained), and RF_Delta_ (λ = 0.0391, 10 features retained). The descriptive statistics (mean, SD, range) and group comparison results (LVI-positive vs. LVI-negative) of all selected radiomics features are presented in Supplementary S1 Table, and all features showed significant differences between the two groups (all p < 0.05) in the training set.

ROC analysis was performed for each selected radiomics feature to evaluate its individual discriminative power for LVI, and the optimal cut-off value was determined by the Youden index. The original_glcm_DifferenceEntropy showed the highest AUC (0.799, cut-off value = 0.586) in RF_Delta_. The wavelet_LHH_firstorder_Kurtosis reached the highest AUC (0.645, cut-off value = 4.4456) in RF_Post_. The wavelet_LLL_glcm_ClusterShade had the highest AUC (0.621, cut-off value = −39674.43) in RF_Pre_. And in RF_T2_, the original_shape_Sphericity had the highest AUC (0.650, cut-off value = 0.634). The ROC curves of all selected radiomics features and their corresponding cut-off values, sensitivity and specificity are shown in Supplementary S1–S4 Fig.

### Construction and Evaluation of Radiomics Signature for LVI Prediction

**Fig 1:**
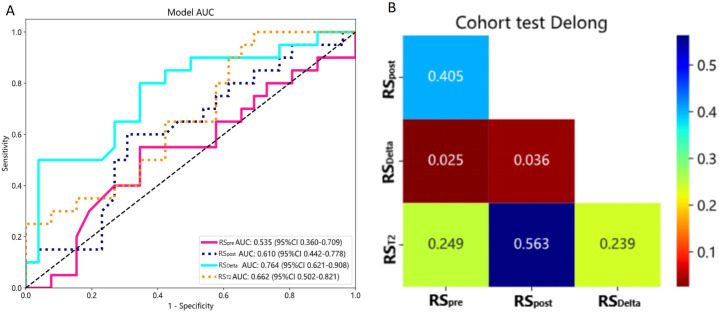
Prediction performance of RS_pre_, RS_post_, RS_T2_, RS_Delta_ in LVI. ROC of Test set (A); Delong test of Test set (B). These showed that RS_Delta_ had the best performance for detecting LVI with AUC of 0.764, which had statistical differences with RS_pre_(*p* = 0.025) and RS_post_ (*p* = 0.036). But ‌‌there was no statistical difference with RS_T2_ (*p* = 0.239).

Four radiomics signatures (RS_T2_, RS_pre_, RS_post_, RS_Delta_) were constructed based on Random Forest model for the selected features of T2FS, pre-contrast, post-contrast and delta-radiomics, respectively. The predictive performance of all signatures in the test set was evaluated with accuracy as the primary metric, and the results are presented in Table 2 and Fig 1.

**Table 2:** Prediction Performance of Various Radiomic Signatures for LVI of Invasive Breast Cancer in Test set.

Radiomic Signatures	Accuracy	AUC(95%CI)	Sensitivity	Specificity	PPV	NPV	*p*
RS_T2_	0.565	0.662(0.502-0.821)	0.500	0.615	0.500	0.615	0.239
RS_pre_	0.565	0.535(0.360-0.709)	0.450	0.654	0.500	0.607	0.025^*^
RS_post_	0.565	0.610(0.442-0.778)	0.350	0.730	0.500	0.594	0.036^*^
RS_Delta_	0.717	0.764(0.621-0.908)	0.500	0.885	0.769	0.697	/

§RS, Radiomic Signatures; * *p* < 0.05; The *p* values obtained from the Delong test, compares the AUCs between RS_Delta_ and RS_T2_, RS_pre_, RS_post_ in the test set.

**Fig 2:**
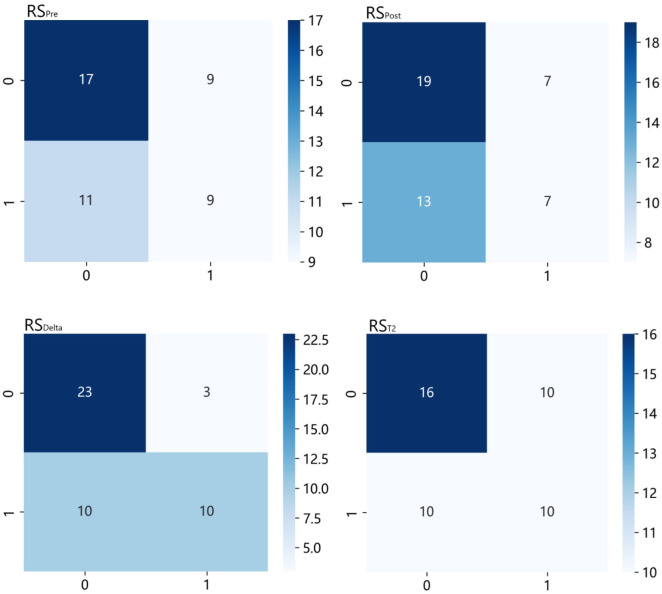
The confusion matrices of the four radiomics signatures in the test set.

In the test set, RS_Delta_ exhibited the best comprehensive performance with an accuracy of 0.717 and AUC of 0.764 (95% CI: 0.621–0.908), followed by RS_T2_ (accuracy = 0.565, AUC = 0.662)，RS_post_ (accuracy = 0.565, AUC = 0.610) and RS_pre_ (accuracy = 0.565, AUC = 0.535). The DeLong test showed that the predictive performance of RS_Delta_ was significantly better than that of RS_pre_ (p = 0.025) and RS_post_ (p = 0.036), while no statistically significant difference was found between RS_Delta_ and RS_T2_ (p = 0.239). The confusion matrices of the four radiomics signatures in the test set are presented in Fig 2. RS_Delta_ showed the highest TN value (23) and the lowest FP value (3) among the four signatures, which was consistent with its highest specificity (0.885).

**Fig 3:**
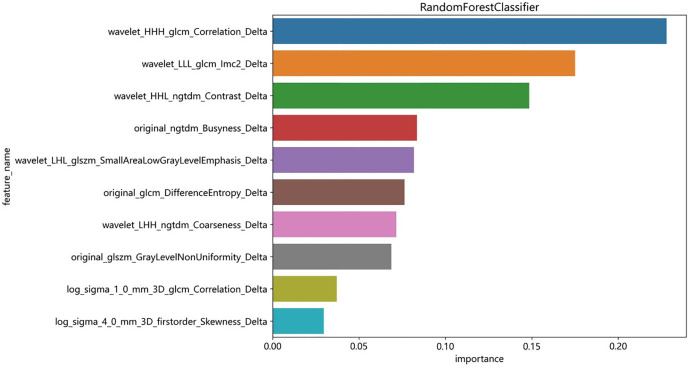
Feature importance of the RS_Delta_ model.

Feature importance of the RS_Delta_ model was calculated and ranked based on the Gini coefficient, and the results are presented in Fig 3. Among the 10 selected RF_Delta_ features, wavelet_HHH_glcm_Correlation (importance score = 0.241),wavelet_LLL_glcm_Imc2 (importance score = 0.189) and wavelet_HHL_ngtdm_Contrast (importance score = 0.151) were the top three most important features for LVI prediction, which contributed the most to the model’s discriminative power.

**Fig 4:**
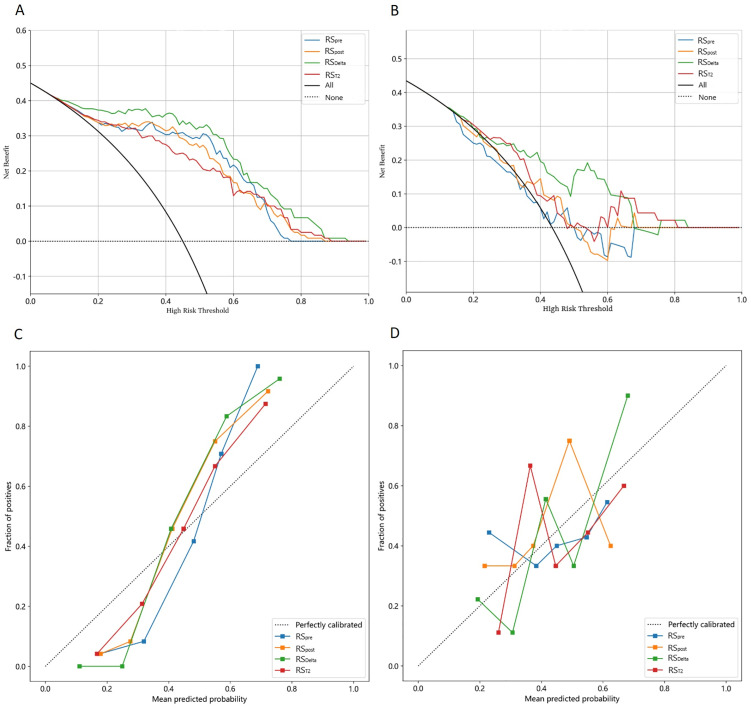
Decision curve analysis(DCA) of four Radiomic Signatures (RS_pre_, RS_post,_ RS_Delta_, RS_T2_) indicating that the RS_Delta_ achieve higher net benefit across most thresholds. **(A)** DCA of Training‌‌ set, **(B)** DCA of Test set, **(C)** Calibration curves of train set, **(D)** Calibration curves of test set.

Decision curve analysis (DCA) was performed to evaluate the clinical net benefit of the four radiomics signatures, and the results are shown in Fig 4A, B. RS_Delta_ achieved a significantly higher net benefit across most threshold probabilities (0.3–0.7) in both the training and test sets compared with RS_T2_, RS_pre_, RS_post_, the “All” strategy (treating all patients as LVI-positive) and the “None” strategy (treating all patients as LVI-negative), which confirmed its high clinical applicability for LVI prediction in invasive breast cancer. The calibration curves of all four signatures in both the training and test sets (Fig 4C, D) showed a good concordance between the predicted LVI probabilities and the actual observed outcomes, indicating good calibration of the models.

## Discussion

### Primary findings and null hypothesis verdict

This study systematically constructed and compared four radiomics signatures based on T2FS, single-phase DCE-MRI and delta-radiomics (DCE-MRI pre- vs. post-contrast) for preoperative LVI prediction in invasive breast cancer. Based on the above study results, the null hypothesis was firmly rejected: the radiomics signatures based on T2FS, pre-contrast DCE-MRI, post-contrast DCE-MRI and delta-radiomics all have significant predictive value for LVI in invasive breast cancer, and there are statistically significant differences in predictive efficacy among these four signatures. Specifically, the delta-radiomics signature (RS_Delta_) showed the most superior predictive performance with a test set accuracy of 0.717 and AUC of 0.764, which was significantly better than the single-phase DCE-MRI signatures (RS_pre_ and RS_post_).

The superior performance of RS_Delta_ is closely associated with the biological characteristics of tumor angiogenesis and LVI. Delta-radiomics features reflect the dynamic changes of tumor microvascular permeability and blood perfusion before and after contrast agent administration, which can more comprehensively capture the abnormal vascular structure of invasive breast cancer (e.g., basement membrane defects, discontinuous endothelial cells) compared with single-phase imaging features. LVI, as a pathological marker of tumor cell invasion into lymphatic or blood vessels, is directly linked to the abnormal angiogenesis of tumors, and the texture and shape features in RS_Delta_ (e.g., wavelet_HHH_glcm_Correlation, wavelet_LLL_glcm_Imc2) can quantify the heterogeneity of tumor vasculature, which is positively correlated with the invasive ability of tumor cells [17–18]. These two features were also identified as the top two most important features in the Random Forest model, which further confirmed the biological rationality of RS_Delta_ for LVI prediction.

Notably, RS_T2_ (AUC = 0.662, accuracy = 0.565) showed no significant difference in predictive performance compared with RS_Delta_ (p = 0.239), which suggests that the peritumoral edema signal captured by T2FS is a potential imaging marker for LVI. T2FS suppresses fat signals to enhance the visualization of peritumoral edema, which is formed due to increased vascular permeability induced by tumor-secreted vascular endothelial growth factor (VEGF) [30–31]. The first-order statistical features (e.g., Skewness) in RS_T2_ can reflect the heterogeneity of water diffusion in edematous regions, which indirectly indicates the aggressiveness of tumor cells and their potential to invade blood or lymphatic vessels [32]. This finding also provides a valuable non-contrast imaging alternative for LVI prediction in patients with renal insufficiency or contrast agent allergy.

### Comparison with existing studies

Previous studies on radiomics-based LVI prediction in breast cancer have mainly focused on single-phase DCE-MRI or T2WI features, with most studies using AUC as the primary evaluation metric and few reporting accuracy, which limits the direct comparison of clinical applicability. In terms of accuracy (the most direct and interpretable metric for clinical practice), the RS_Delta_ in this study achieved a test set accuracy of 0.717, which is comparable or superior to the results of previous relevant studies. Liu et al. [17] constructed a post-contrast DCE-MRI radiomics model with an AUC of 0.78 and an unreported accuracy for LVI prediction, while the RS_post_ in this study showed a relatively low AUC (0.610) and accuracy (0.565), which may be due to the larger sample size (166 vs. 112 cases) and stricter feature selection (mRMR + LASSO) in our study. Zheng et al. [16] first proposed the delta-radiomics concept for LVI prediction and reported an AUC of 0.79 with an accuracy of 0.72 in their study, which is highly consistent with our results (AUC = 0.764, accuracy = 0.717), and this consistency further validates the universality and reliability of delta-radiomics for LVI prediction in invasive breast cancer.

In addition, our study further analyzed the feature importance of the Random Forest model and identified the key radiomics features for LVI prediction, which not only provides a basis for the interpretation of the black box model but also lays a foundation for the subsequent development of targeted imaging biomarkers.

### Clinical translation value and application scenarios

The high predictive performance and good clinical net benefit of RS_Delta_ make it a potential non-invasive imaging biomarker for preoperative LVI prediction in invasive breast cancer, and its high specificity (0.885) is particularly valuable for clinical decision-making. For patients with a negative RS_Delta_ prediction, the risk of LVI is low, and clinicians can consider a reduced surgical scope (e.g., breast-conserving surgery instead of mastectomy) or avoid unnecessary adjuvant chemotherapy, which can reduce the physical and economic burden on patients. For patients with a positive RS_Delta_ prediction, intensified adjuvant therapy (e.g., chemotherapy, targeted therapy) can be administered in a timely manner to reduce the risk of disease recurrence and metastasis [8,13]. Successful clinical translation requires rigorous validation, regulatory compliance, and integration into routine radiology workflow [23].

In addition, the moderate predictive performance of RS_T2_ provides an important non-contrast imaging alternative for LVI prediction, which fills the clinical gap for patients who cannot receive contrast-enhanced MRI due to renal insufficiency, contrast agent allergy or other reasons. Decision curve analysis showed that RS_Delta_ has a higher net benefit than traditional clinical features (e.g., tumor diameter, ALN status) when the clinical threshold probability exceeds 0.3, which indicates that RS_Delta_ can be used as an independent risk stratification tool for invasive breast cancer and combined with clinical and molecular features to construct a multi-modal prediction model for more accurate LVI prediction [33].

### Clinical deployment, regulatory challenges, device heterogeneity, and ethical implications

Before clinical translation, several critical issues must be fully addressed for real‑world deployment. First, clinical deployment requires integration of the RS_Delta_ model into routine PACS or dedicated AI-assisted diagnostic platforms, with a standardized workflow including automatic image preprocessing, tumor segmentation, feature calculation, and LVI risk output. The model is intended for preoperative risk stratification in treatment‑naïve invasive breast cancer patients, not as a replacement for histopathological diagnosis.

Second, regulatory challenges involve compliance with medical AI regulations (e.g., NMPA, FDA) for software as a medical device (SaMD). Prospective validation, algorithm transparency, quality control protocols, and audit trails are mandatory for clinical certification [22].

Third, device heterogeneity remains a key obstacle. This study used 1.5T and 3.0T scanners from multiple vendors with sequence standardization and resampling; however, broader application requires cross‑vendor calibration, domain adaptation, or federated learning to maintain performance without sharing raw patient data [23,24].

Fourth, ethical implications include data privacy, informed consent, and algorithmic fairness. All data were de‑identified and approved by the ethics committee; future applications will uphold patient autonomy, avoid bias across subgroups, and clearly communicate the predictive nature of the model to clinicians and patients [25].

### Limitations and future directions

This study still has several limitations that need to be addressed. First, it is a single-center retrospective study, and the LVI positivity rate in the sample (45% in the training set, 43% in the test set) is higher than the 20%−30% reported in the literature [13], which may introduce selection bias. Future multicenter, prospective studies with larger sample sizes and balanced LVI positivity rates are needed to further validate the performance of the RS_Delta_ signature. Second, manual ROI delineation was used for tumor segmentation, which has a certain degree of subjectivity despite the screening of features with ICC > 0.75 and independent segmentation by two radiologists. The introduction of automated segmentation algorithms (e.g., deep learning-based semantic segmentation) in future studies can further improve the objectivity and reproducibility of feature extraction [29]. Third, this study only included patients who underwent surgical resection without neoadjuvant chemotherapy, and the predictive value of delta-radiomics for LVI in patients receiving neoadjuvant chemotherapy remains to be explored.

Future research can be expanded in the following directions: (1) To improve generalizability, we propose a three‑stage external, multicenter, and prospective validation plan: a.External validation: The RS_Delta_ model will be tested on independent retrospective cohorts from two or more external medical centers. b.Multicenter validation: A combined multi‑institutional dataset will be used to optimize model stability across scanners, protocols, and populations. c.Prospective validation: A prospective observational study will be initiated to enroll consecutive preoperative breast cancer patients and evaluate real‑time diagnostic performance in clinical practice; (2) Include patients receiving neoadjuvant chemotherapy to evaluate the value of delta-radiomics in monitoring treatment efficacy and predicting post-therapy LVI status; (3) Develop a mobile AI-assisted diagnostic app based on the RS_Delta_ signature to translate the radiomics model into bedside clinical tools, which can improve the LVI prediction ability of primary hospitals and promote the individualized treatment of invasive breast cancer. Recent advances in biomedical artificial intelligence, including explainable AI, federated learning, and clinical translation, have provided robust methodological support for radiomics research and its real-world implementation [20,21,23,24].

## Conclusion

This study confirms that the delta-radiomics signature (RS_Delta_) based on DCE-MRI dynamic changes has significant predictive value for LVI in invasive breast cancer, with a test set accuracy of 0.717 and high specificity, which is significantly superior to the single-phase DCE-MRI signatures and comparable to the T2FS signature. The T2FS signature provides a valuable non-contrast imaging alternative for LVI prediction in clinical practice. These non-invasive and reproducible radiomics signatures can serve as important supplements to traditional histopathological examination, provide a reliable basis for preoperative risk stratification and individualized treatment decision-making of invasive breast cancer, and have important clinical translation value. Future multicenter, prospective studies and radiogenomics research are ‌‌needed to further validate and optimize these models.

## Supporting information

S1 Fig ROC curves of individual radiomic features in the T2FS model for LVI prediction.
(TIF)

S2 Fig ROC curves of individual radiomic features in the pre-contrast DCE-MRI model for LVI prediction.
(TIF)

S3 Fig ROC curves of individual radiomic features in the post-contrast DCE-MRI model for LVI prediction.
(TIF)

S4 Fig ROC curves of individual radiomic features in the delta-radiomics model for LVI prediction.
(TIF)

S1 Table Descriptive statistics and group comparisons of the selected radiomic features.
(XLSX)
