# Supplementary figures and images for: Machine learning models based on magnetic resonance imaging for predicting Lymphovascular Invasion in Invasive Breast Cancer
Source: PLoS One. 2026 May 29;21(5):e0350085. doi: 10.1371/journal.pone.0350085 (PMC13221042; doi:10.1371/journal.pone.0350085)

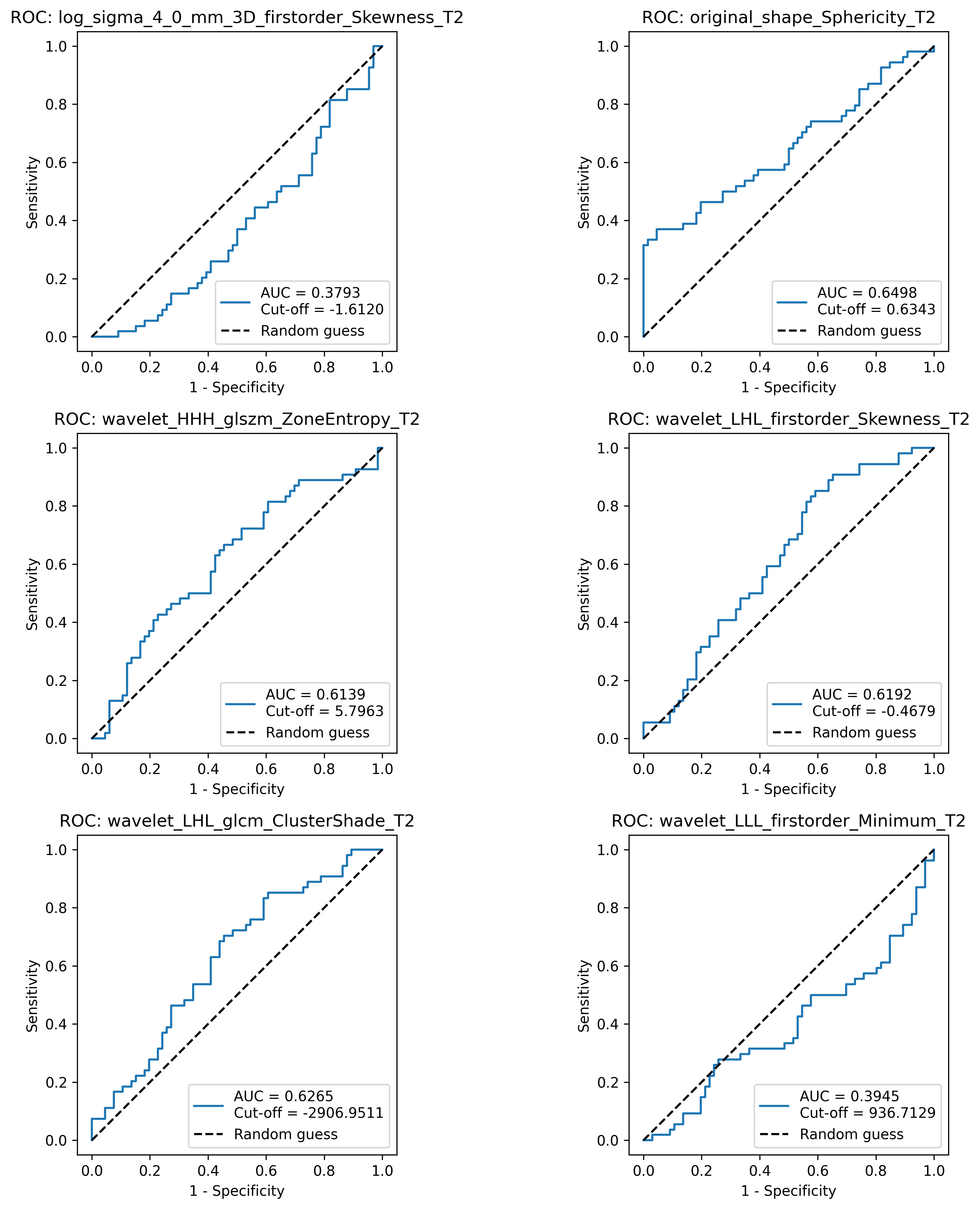

Supplement: S1 Fig — ROC curves of individual radiomic features in the T2FS model for LVI prediction. (TIF) [file pone.0350085.s001.tif]

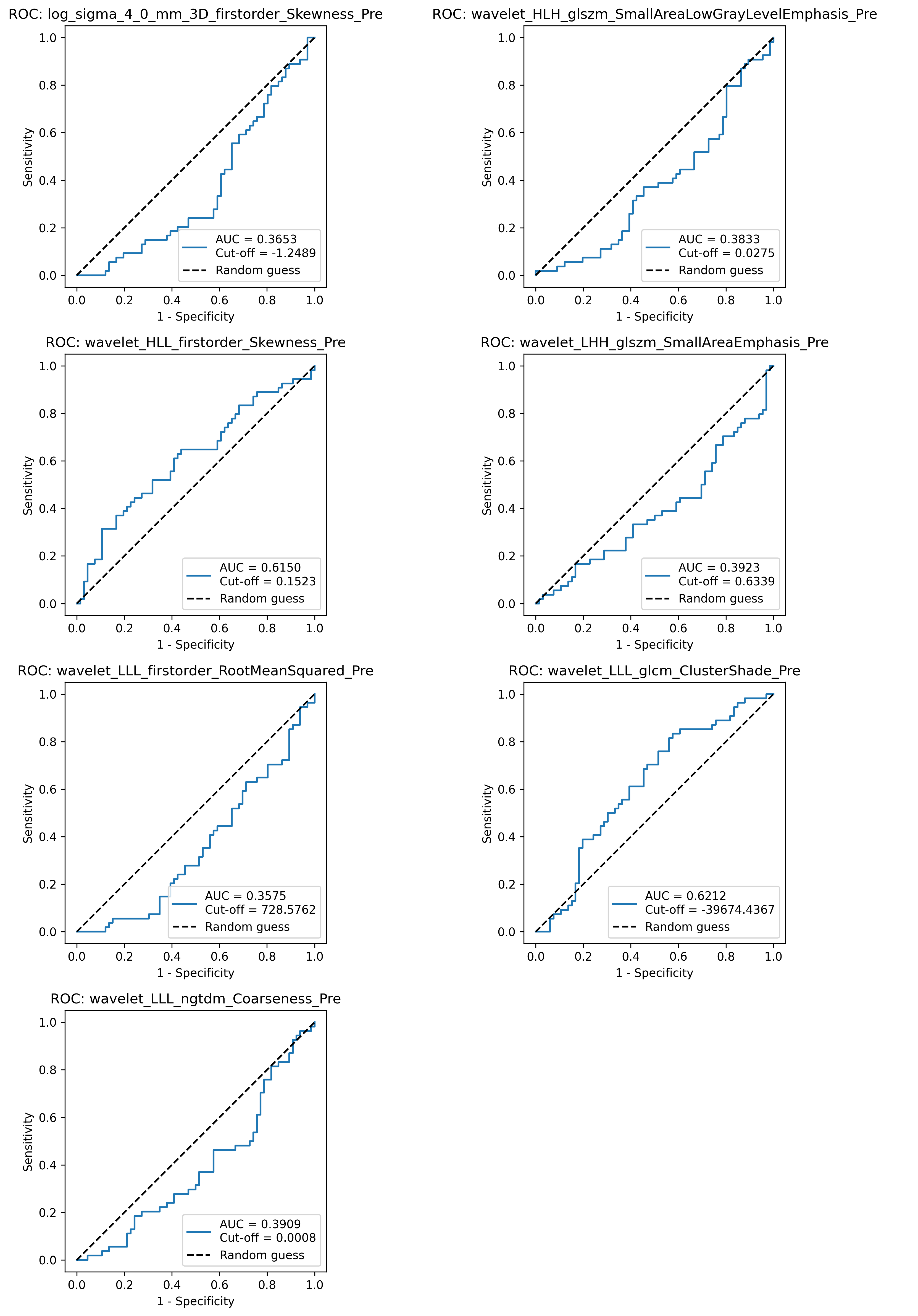

Supplement: S2 Fig — ROC curves of individual radiomic features in the pre-contrast DCE-MRI model for LVI prediction. (TIF) [file pone.0350085.s002.tif]

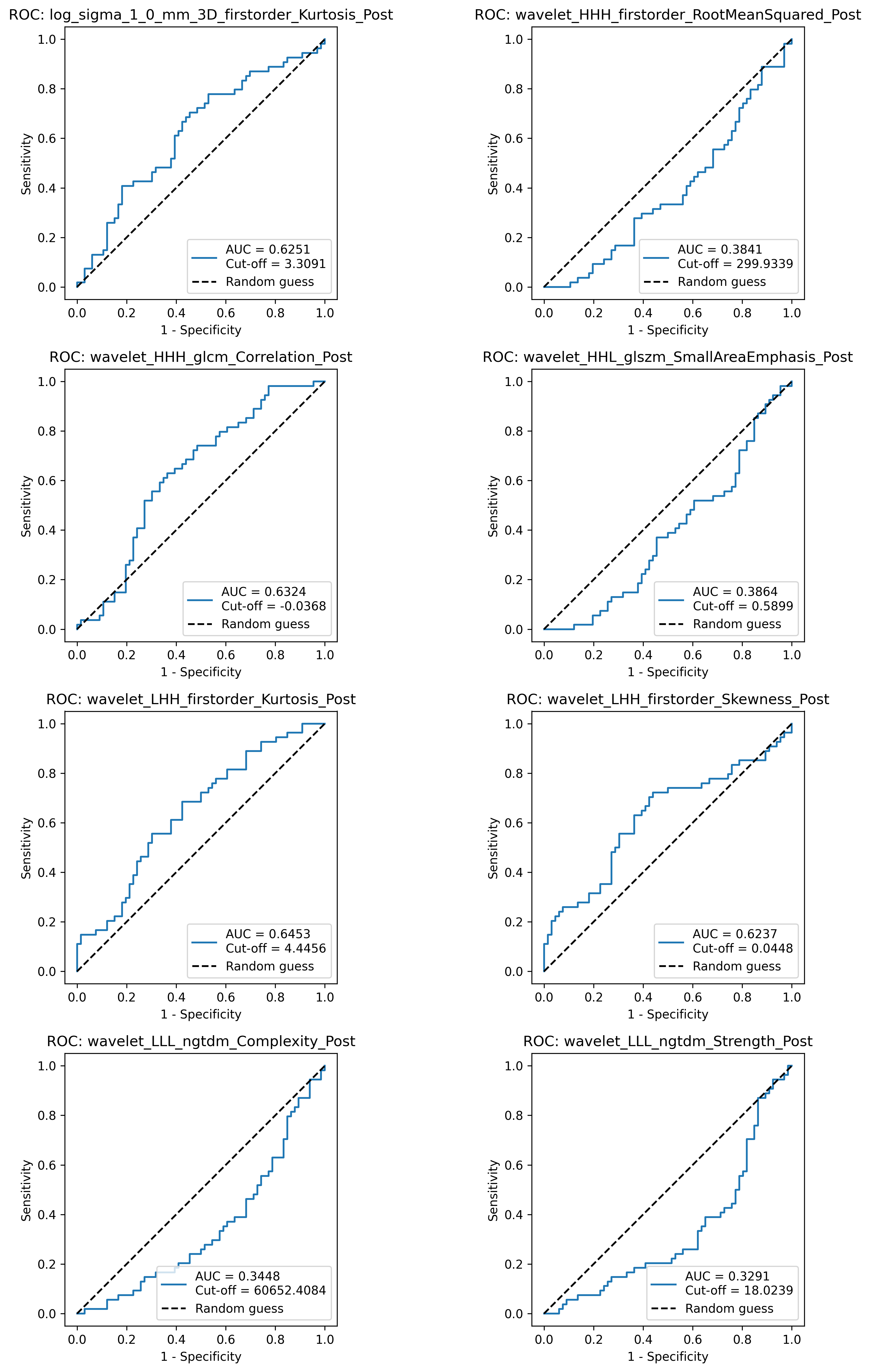

Supplement: S3 Fig — ROC curves of individual radiomic features in the post-contrast DCE-MRI model for LVI prediction. (TIF) [file pone.0350085.s003.tif]

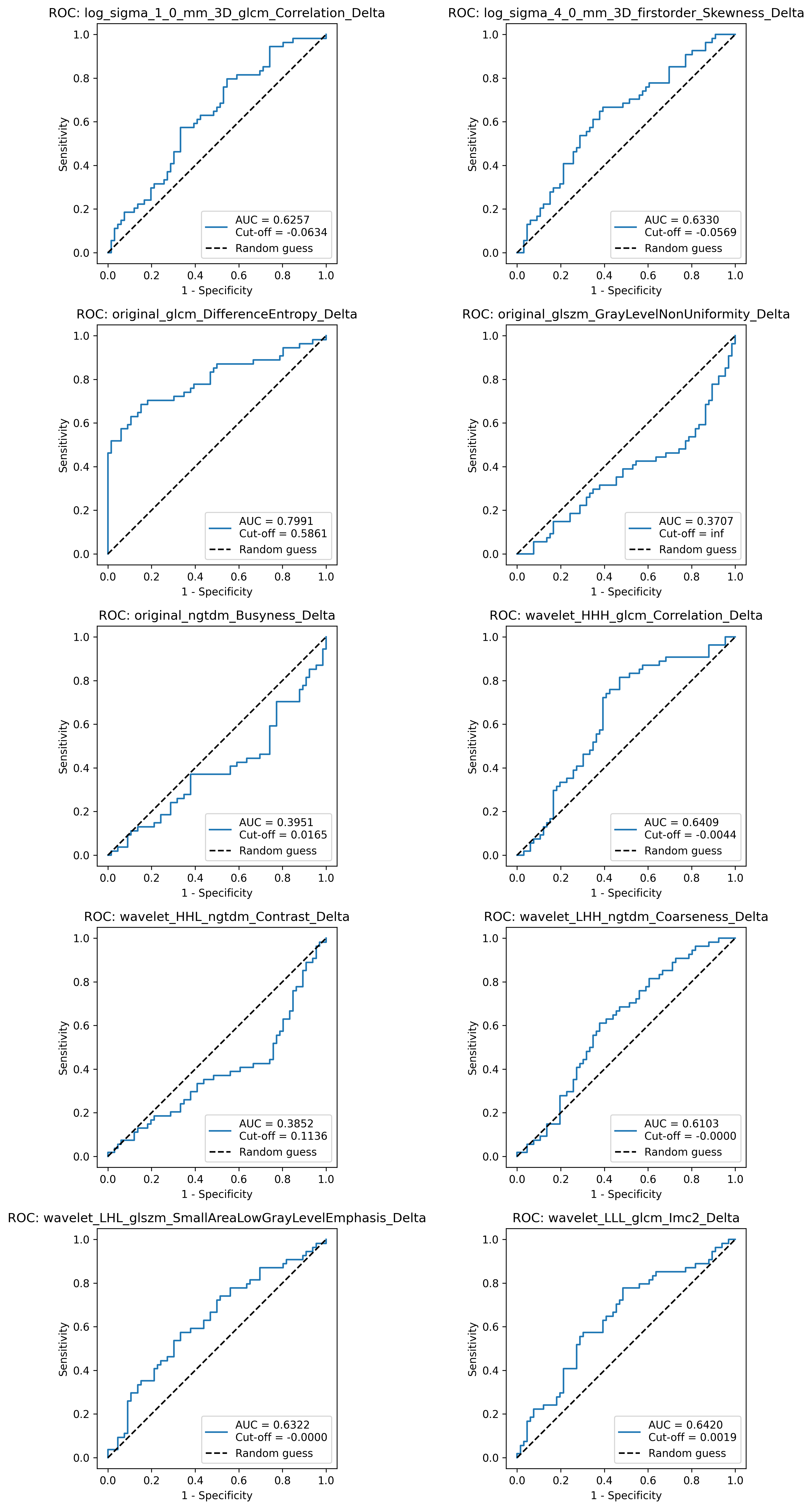

Supplement: S4 Fig — ROC curves of individual radiomic features in the delta-radiomics model for LVI prediction. (TIF) [file pone.0350085.s004.tif]
